# Evaluation of psychological distress is essential for patients with locally advanced breast cancer prior to neoadjuvant chemotherapy: baseline findings from cohort study

**DOI:** 10.1186/s12905-023-02571-1

**Published:** 2023-08-23

**Authors:** Majid Omari, Lamiae Amaadour, Btissame Zarrouq, Yazeed A. Al-Sheikh, Achraf El Asri, Salima Kriya, Sara Nadi, Zineb Benbrahim, Nawfel Mellas, Karima El Rhazi, Mohammed El Amine Ragala, Jaouad El Hilaly, John P. Giesy, Mourad A.M. Aboul-Soud, Karima Halim

**Affiliations:** 1 Laboratory of Epidemiology and Research in Health Sciences, Faculty of Medicine and Pharmacy, Sidi Mohamed Ben Abdallah University, Fez, Morocco; 2 Laboratory of Natural Substances, Pharmacology, Environment, Modeling, Health & Quality of Life, Faculty of Sciences Dhar El Mahraz, Sidi Mohamed Ben Abdellah University, Fez, Morocco; 3 Department of Medical Oncology, Hassan II University Hospital, Fez, Morocco; 4 Department of Biology and Geology, Teacher’s Training College (Ecole Normale Supérieure), Sidi Mohamed Ben Abdallah University, Fez, Morocco; 5 Department of Clinical Laboratory Sciences, College of Applied Medical Sciences, King Saud University, P.O. Box 10219, Riyadh, 11433 Saudi Arabia; 6 Higher Institute of Nursing Professions and Health Techniques, Fez, Morocco; 7 Laboratory of Pedagogical and Didactic Engineering of Sciences and Mathematics, Regional Center of Education and Training (CRME F) of Fez, Fez, Morocco; 8 R.N.E Laboratory, Multidisciplinary Faculty of Taza, Sidi Mohamed Ben Abdellah University, Fez, Morocco; 9 Toxicology Centre, University of Saskatchewan, Saskatoon, SK S7N 5B3 Canada; 10 Department of Veterinary Biomedical Sciences, University of Saskatchewan, Saskatoon, SK S7N 5B4 Canada; 11 Department of Integrative Biology, Michigan State University, East Lansing, MI 48824 USA; 12 Department of Environmental Sciences, Baylor University, Waco, TX 76706 USA; 13 Department of Human and Social Sciences - Education Sciences, Teachers Training College (Ecole Normale Superieure), Sidi Mohamed Ben Abdellah University, Fez, Morocco

**Keywords:** Psychological distress, Depression, Anxiety, Sociodemographic factors, Clinical factors, Locally advanced breast cancer, Neoadjuvant chemotherapy

## Abstract

patients with locally advanced breast cancer (LABC) are often treated by neoadjuvant chemotherapy (NACT). This study aims to determine the prevalence of psychological distress and their sociodemographic and clinical factors in patients recently diagnosed with LABC and before NACT. Methods: A total of 209 LABC patients without metastatic localization were recruited between 2021 and 2022 in the oncology hospital at Fez. A structured questionnaire and the Hospital Anxiety and Depression Scale were used. A descriptive analysis and linear model were performed. Results: a mean age of participants was 47.43 ± 9.45 years. The prevalence of depression, anxiety and psychological distress among participants was 59.62% (95% CI: 52.61–33.34), 47.85% (95% CI: 40.91–54.85), and 65.07 (95% CI: 58.19–71.52) respectively. Depression was associated to age (< 50 years) (AOR = 2.19; 95% CI = 1.13–4.23) and health insurance (AOR = 3.64; 95% CI: 1.18–11.26). Anxiety was associated to age (< 50) (AOR = 2.21; 95% CI: 1.18–4.13) and right breast cancer (AOA = 2.01; 95% CI: 1.11–3.65). Psychological distress was associated to chronic illness (AOR = 2.78; 95% CI: 1.32–5.85) and lymph node status (AOR = 2.39; 95% CI: 1.26–4.57). Conclusions: Based on the high prevalence of depression and anxiety it appears opportune to treat psychological distress among LABC patient candidates for NACT. Each psychological intervention should take into account sociodemographic and clinical factors found associated in our study. Psychological therapeutic interventions are crucial for LABC patients as early as the time of diagnosis and through subsequent steps in NACT to improve their overall mental health.

**Supplementary Information:**

The online version contains supplementary material available at 10.1186/s12905-023-02571-1.

## Introduction

The global cancer statistics report states that the number of new cases of breast cancer (BC) was approximately 2.3 million in 2020 [1] and it is the most common malignancy among women globally, accounting for 30% of new cancers [2]. Compared to other tumors, BC responds better to treatment and patients are more likely to survive five years [3].

The experience of BC or its treatment can be traumatic for patients due to its impact on their sexual relationship and self-image. Patients suffered from physical, psychosocial, and spiritual problems. Consequently, most BC patients have psychological reactions, including anger, denial or fear related to the disease and treatment process, and many of them have mental illnesses [4–6]. It has been reported that 50% of BC patients experience clinically psychological distress because of their disease [7]. Having psychological distress hinders receiving necessary follow-up care, potentially resulting in an increased mortality [8].

According to National Comprehensive Cancer Network (NCCN) [9] Guidelines for Managing Distress, psychological distress is considered as “a multifactorial and unpleasant experience of a psychological (i.e., cognitive, behavioral, emotional), social, spiritual nature; and/or physical which may interfere with the ability to effectively cope with cancer, its physical symptoms and treatment”. Psychological distress is a construct that covers psychiatric symptoms such as depression and anxiety. Psychological distress has significant overlap with anxiety and depression concepts, which have been widely used as psychological disorder related terms [10–16]. As a result of unpleasant stimuli, anxiety is defined as an intense feeling of apprehension, excessive fear and uncertainty. There are multiple dimensions to anxiety, including cognitive, physiological, and physical aspects [17]. While, depression is characterized by changes in mood and cognition over a period of at least two weeks as well as a loss of interest or pleasure [18].

Depression and anxiety and are the most common mental illness. Patients with BC may experience symptoms of depression and anxiety which affect their physical and psychological health, and may even pose a significant risk factor for mortality [19, 20]. According to two recent systematic reviews and meta-analyses, there are high levels of depression and anxiety among BC patients worldwide, 32.2 and 41.9%, respectively [19, 20]. Studies investigating psychological adjustment have showed different outcomes if they were evaluated patients who had recently been diagnosed or going through treatment, as well as who had already completed the principal steps of care [7, 16, 21]. Moreover, Yang [22] reported an augmented risk of mental disorders immediately following a BC diagnosis, suggesting the need to research both diagnosis and treatment separately.

Since BC is a chronic disease, it is crucial to address how patients adapt to it and identify what factors help them thrive. Psychological adjustment can be viewed as a complex concept, although there are several definitions. Recently, some authors have advocated taking a more global and multidimensional approach. Larsen and Hummel [23] discussed that adjustment to cancer involves not only managing medical and physical challenges associated with cancer, but also addressing dimensions of emotional, cognitive, behavioral, and psychological functioning. Psychological adjustment studies have shown different results depending on whether they were conducted on patients with a recently BC diagnosed, during therapy or with patients who had completed treatment [7, 16, 21].

In low and middle-income countries, 40 to 60% of all BC diagnosed are LABC [24–26], while it accounts for only 4–8% of all of BC women in the Europe and United States [25]. Sub-Saharan African BC patients are more of risk to be presented at diagnosis with advanced stage, which leads to poor survival rates, according to a systematic review [27]. Approximately 40% of BC in Saudi Arabia was LABC at time of diagnosis in 2015 [28]. A similar picture applies to Morocco, where most women begin treatment for BC at advanced stages [29]. A published study showed that between 2008 and 2017, the rate of women diagnosed with advanced BC was about 45% [30]. Nevertheless, these statistics remain questionable since they might include cases with metastases of BC and it is not defined uniformly across centers [31], there is therefore a need to define LABC.

In fact, the Union for International Cancer Control (UICC) and the American Joint Committee on Cancer (AJCC) staging systems usually define LABC as stage IIIB or IIIC, but some clinicians include patients with stage IIIA disease as LABC. Inflammatory BC is also included in LABC [4, 32]. Breast tumours larger than 5 cm without regional lymphadenopathy have also been included as LABC by some authors (T3N0M0) [33–36].” In the treatment of LABC, neoadjuvant therapy is usually recommended to downstage the disease, followed by timely surgery, radiotherapy, and systemic therapy following the surgery [24, 26, 37, 38]. The purpose of NACT is to ameliorate surgical options and to assess treatment response in the breast. In addition, NACT allows for the discovery of predictive markers of chemotherapy [39].

In light of the large number of Moroccan patients with LABC, we consider it useful to investigate their psychological determinants. Thus, we are conducting a cohort study to understand the psychological adjustment of Moroccan patients with LABC undergoing neoadjuvant chemotherapy and to determine how psychological distress evolves through the course of their treatment. Therefore, from this cohort study, the main objective of this article is to assess the prevalence of psychological distress, depression and anxiety and their sociodemographic and clinical determinants in Moroccan women with LABC newly receiving diagnosis and waiting NACT.

## Materials and methods

### Study design

This was a cross-sectional study conducted on the baseline cohort. The aim of the study was to investigate the prevalence and determinants of psychological distress, depression, and anxiety among women newly diagnosed with LABC and who are candidates for NACT. This study was approved by the hospital-university ethics committee related to University of Sidi Mohamed Ben Abdellah (N°24/18). It was conducted at the public oncology hospital of Fez city. A total of 209 patients with BC who were diagnosed with LABC between 2021 and 2022, were recruited during the first medical oncology consultations and before starting NACT.

Inclusion criteria were: (i) women over 18; (ii) women who had been diagnosed with BC, in absence of distance metastases and should start NACT; iii); women who had signed an informed consent to take part in the study. Exclusion criteria were: women with a history of psychiatric disease prior to be diagnoses with BC; and women with other malignancies.

### Sociodemographic and clinical variables

Sociodemographic variables recorded were age at diagnosis, marital status, having children, number of children, education, profession, health insurance coverage, residency, ethnicity, patient’s monthly income, and monthly family income. Two age categories (> 50 years, ≤ 50 years) were created. Marital status was grouped into two categories (married/widow, divorced or unmarried). Residential location was dichotomized into urban/rural. Patients were asked if they have children (yes/no) and their number (< 3, ≥ 3), while two categories were created for profession (Unemployed/employed). Women were also classified according to their Arab or Amazigh ethnic origin. Level of education was categorized into two groups (illiterate vs. educated). Health insurance coverage was divided into two categories (total and partial). Finally, patients were classified according to their monthly income (No income/ income ≤ 250$), and household income (< 250$/ $$\ge$$250 and < 500$).

The present study included clinical variables such as the breast tumor laterality (right/left), menopause status (pre/post), time from finding out the first symptoms, family cancer history (yes/no), and the presence of other chronic illness (yes/no).

In addition, histological type was added as a variable and categorized as ductal/lobular and others type. As part of the Tumor-Node-Metastasis (TNM) classification, we also included tumor size (T1 or T2/ T3) and lymph node status (negative/positive). The estrogen receptor (ER) progesterone receptor (PR) was considered positive if they were present in 10% or more of the cells and human epidermal growth factor receptor 2 (HER2). The Ki67 labeling index was 20%. For molecular types, luminal A (ER + and/or PR+, HER2-), luminal B (ER + and/or PR+, HER2+), HER2 enriched (ER-, PR-, HER2+), and triple-negative breast cancer (TNBC) (ER-, PR-, HER2-) were searched.

### Hospital anxiety and depression scale

Psychological distress of our participants was assessed by the Hospital Anxiety and Depression Scale (HADS), established in by Zigmond et al. [40]. The HADS is a 14-item scale that measures depression and anxiety with a high score of 21 for each one. The summarized minimum score of each of the seven item subscales is 07 and the maximum 21. The HADS total score showed good screening utility to detect the presence of distress in an oncology setting [41, 42]. The cutoff used was $\ge 10$ units to categorize anxiety caseness (HADS-A), and $\ge 8$ units for depression (HADS-D). According to several papers, we used HADS total score of ≥15 as cutoff for overall psychological distress [42–51].

A HADS was administered to our study subjects before they received the NACT. The reliability and validity of the Arabic version of HADS were established in a previous study [52]. In this study, HADS had high internal consistency (Cronbach’s α = 0.91).

### Statistical analyses

According to Epitools epidemiological calculators, the minimal sample of 160 was deemed necessary. In neoadjuvant trials, the best pathological complete response is around 50% and the best absolute difference is around 20% [53, 54]. Then, the estimated best relative risk for improvement in pathological complete response (PCR) after neoadjuvant chemotherapy is close to 1.7 [55]. Based on passive coping behavior, we can estimate a PCR rate of 30%, while an active coping behavior will have a relative risk of 1.7. thus, the active coping behavior will have a 50% PCR rate. The power of the study would be 80% and the p-value would be 0.05. More than 40% is added to replace patients who were lost to follow-up, patients whose NACT protocol changed as a result of the progression of the disease, patients who died during the course of NACT, patients who left the hospital for another care hospital, and patients who refused to sign consent forms or refused follow-up within the cohort framework. As a result of this cohort study, a total of 209 participants were recruited at baseline.

Data were entered using Epi Info version 7.2.3.1 and was analyzed statistically using SPSS version 25.0 and psych package in R computing environment (4.1.1) for analyzing the general description of the questionnaires results and comparison of the score. Categorical variables were represented in percentages and frequencies. Continuous variables were presented in means and standard deviation because they respected normal distribution with its skewness and kurtosis varying between − 1 and + 1. Chi-square test (χ^2^) and Fisher’s exact test were operated to describe distributions of anxiety, depressive and psychological distress in categorical sociodemographic and clinical variables. A confidence interval of 95% was achieved with 0.05 level of significance. A multivariate logistic regression analysis was performed including selected correlates with *p* < 0.2 in bivariate analysis. The results were reported as adjusted odds ratio with 95% CI.

## Results

A total of 209 BC patients in this study, their mean age was 47.43 ± 9.45 years, with 70.81% were married and most of them have children (89.71%). The majority (87.56%) are benefiting from a total health insurance coverage, since 87.08% are unemployed and 89.00% are without monthly income. Other sociodemographic characteristics are summarized (Table 1).

**Table 1:** Sociodemographic variables of the sample (n = 209)

Sociodemographic Variables	Percentage (%)
**Age at diagnosis**	
< 50	61.24
$$\ge$$ 50	38.76
**Marital status**	
Married	70.81
widow/divorced, unmarried	29.19
**Having children**	
No	10.29
Yes	89.71
**Number of children**	
< 3	44.57
≥ 3	55.43
**Education**	
Illiterate	63.16
Educated	36.84
**Profession**	
Unemployed	87.08
Employed/	12.92
**Health insurance coverage**	
Total	87.56
Partial	12.44
**Residency**	
Rural	32.06
Urbain	67.94
**Ethnicity**	
Amazigh	24.88
Arabic	75.12
**Patient’s monthly income**	
No income	89.00
≤ 250$	11.00
**Monthly Family income**	
No income	15.61
< 250$	71.71
$$\ge$$250 and < 500$	12.68

For clinical variables, 61.72% of participants in the present study belonged to T4, while 38.28% were diagnosed with T2 or T3, moreover, 62.20% had a positive lymph node status. Laterality of BC was right in 51.67% of cases. The most common histological type of BC was invasive ductal carcinoma in 93.27%. For hormone receptors, ER was positive in 60.19%, and PR was positive in 49.76% of patients. Among molecular subtypes, we noted that 6.06%, 35.86%, 20.71% and 12.12% had respectively Luminal A, Luminal B, Luminal B HER2 and HER2 enriched, and that 25.25% has TNBC. Other clinical variables are presented in Table 2.

**Table 2:** Clinical variables of the sample (n = 209)

Clinical variables	Percentage (%)
**Laterality**	
Left	48.33
Right	51.67
**Menopause status**	
Premenopausal	50.24
Postmenopausal	49.76
**Time from finding out the first symptoms**	
< 6 months	44.00
≥ 6 and < 12 months	26.29
≥ 12 months	28.71
**Family Cancer History**	
No	85.17
Yes	14.83
**Chronic illness**	
No	78.37
Yes	21.63
**Histological type**	
Ductal	93.27
Lobular and others	6.73
**Tumour size**	
T2/T3	38.28
T4	61.72
**Lymph node status**	
Negative	37.80
Positive	62.20
**Molecular subtype**	
Luminal A	6.06
Luminal B	35.86
Luminal B HER2	20.71
HER2^1^ enriched	12.12
Triple negative	25.25
**ER** ^2^	
Negative	39.81
Positive	60.19
**PR** ^3^	
Negative	50.24
Positive	49.76
**HER2**	
Negative	66.50
Positive	33.50
**KI 67**	
< 20	13.23
≥ 20	86.77

^1^ Human epidermal growth factor receptor-2, ^2^ Estrogen receptor,^3^ Progesterone receptor.

Among the 209 participants, 59.62% (95% CI: 52.61–33.34) (n = 125) women were found to be depressed, 47.85% (95% CI: 40.91-54.85%) (n = 100) were anxious and 65.07% (95% CI: 58.19–71.52) (n = 135) were suffering from psychological distress (Table 3).

**Table 3:** Prevalence of psychological distress, depression and anxiety among participants (n = 209)

Psychological variables	Percentage (95% CI^1^)	Mean ± SD^2^	
**Anxiety**		9.04 ± 4.83	
Negative	52.15 (45.15–59.09)		
Positive	47.85 (40.91–54.85)		
**Depression**		8.42 ± 4.33	
Negative	40.38 (33.66–47.39)		
Positive	59.62 (52.61–33.34)		
**Psychological distress**		17.46 ± 8.70	
Negative	34.93 (28.48–42.81)		
Positive	65.07 (58.19–71.52)		

^1^ Confidence interval, ^2^ standard deviation

A bivariate analysis was conducted to assess the association between anxiety, depression, and psychological distress, by the Chi-square test and the Fisher’s exact test. Based on the Supplementary Information (Additional file 1: Supplementary Table S1), the monthly income of the family (χ2 = 7.87, p = 0.01), and the laterality of BC (χ2 = 6.67, p = 0.01; OR = 0.48) were significantly associated with anxiety. Patients with another chronic illness (χ2 = 5.34, p = 0.02; OR = 0.45) and lymph node status (χ2 = 5.34, *p* = 0.01; OR = 0.48) were associated with depression. It was showed also that psychological distress was associated to age (χ2 = 3.99, *p* = 0.05; OR = 0.55), other chronic illness (χ2 = 6.46, *p* = 0.01; OR = 0.42), positive lymph node status (χ2 = 7.92, *p* = 0.007; OR = 2.30)) and molecular subtypes (χ2 = 12.14, *p* = 0.01) (Supplementary Table S1).

Logistic regression analyzes were conducted to examine the associations of sociodemographic and clinical factors with depression, anxiety and psychological distress in patient newly diagnosed with LABC and before receiving NACT (Table 4).

**Table 4:** Univariate and multivariate regression analysis of the association between sociodemographic and clinical variables with depression, anxiety, and psychological distress

	Anxiety		Depression		Psychological distress	
	COR^4^ ((95%CI^5^)	AOR^6^ (95%CI)	COR (95%CI)	AOR (95%CI)	COR (95%CI)	AOR (95%CI)
**Age**						
≥ 50	1	1	1	1	1	
< 50	0.57*(0.32–1.01)	2.21*(1.18–4.13)	1.55 (0.88–2.74)	2.19* (1.13–4.23)	1.80*(1.01–3.22)	
**Lymph node status**						
Negative	1		1		1	1
Positive	1.75*(0.99–3.09)		1.82*(1.02–3.22)		2.30**(1.28–4.13)	2.39**(1.26–4.57)
**Chronic illness**						
No	1		1		1	1
Yes	1.66 (0.84–3.27)		0.45*(0.23–0.89)		2.36*(1.20–4.63)	2.78**(1.32–5.85)
**Molecular subtype**						
Luminal A	1		1		1	
Luminal B	0.58 (0.17–2.02)		0.35 (0.08–1.41)		0.24 (0.05–1.19)	
Luminal B HER2	0.45 (0.12-1 ;69)		0.38 (0.09–1.63)		0.28 (0.05–1.45)	
Her2 Enriched	0.42 (0.10–1.76)		0.66 (0.14–3.16)		0.40 (0.07–2.27)	
TNBC	1.16 (0.32–4.19)		0.77 (0.18–3.28)		0.91 (0.17–4.89)	
**Health insurance coverage**						
Partial			1	1	1	
Total			2.05 (0.88–2.74)	3.64*(1.18–11.26)	2.05 (0.89–4.69)	
**Breast Cancer Laterality**						
Left	1	1			1	
Right	0.48**(0.27–0.84)	2.01*(1.11–3.65)			0.61 (0.34–1.09)	
**Nomber of childern**						
< 3			1			
≥ 3			1.56 (0.84–2.90)			
**Having children**						
No			1			
Yes			0.40 (0.12–1.27)			
**Monthly family income**						
No income	1		1			
< 250$	0.33**(0.14–0.75)		0.34 (0.14–0.85)			
Between 250$ and 500$	0.53 (0.18–1.55)		0.38 (0.12–1.19)			
**ER** ^**1**^						
Negative			1		1	
Positive			0.62 (0.35–1.12)		0.58 (0.31–1.06)	
**PR** ^**2**^						
Negative					1	
Positive					0.56 (0.31-1.00)	
**HER2** ^**3**^						
Negative	1					
Positive	0.59 (0.33–1.07)					
**Tumour size**						
T2/T3			1			
T4			1.48 (0.84–2.61)			

*p ≤ 0.05; **p ≤ 0.01, ^1^Estrogen receptor, ^2^Progesterone receptor; ^3^Human epidermal growth factor receptor 2, ^4^Cured Odds Ratio, ^5^Confidence interval, ^6^Adjusted Odds Ratio

Regarding depression, multivariate analysis confirmed the result of univariate analysis, patients under 50 years old are 2.19 times higher risk to suffer from depression than those over 50 years of age (AOR = 2.19; 95% CI = 1.13–4.23). In addition, those with full health insurance are 3.64 times greater risk of depression than those with partial coverage (AOR = 3.64; 95% CI: 1.18–11.26) (Table 4).

Concerning anxiety, multivariate analysis revealed its association with age less than 50 years old (AOR = 2.21; 95% CI: 1.18–4.13) and right side as BC laterality (AOR + 2.01, 95% CI: 1.11–3.65) (Table 4).

For psychological distress, univariate binary logistic regression analysis showed that it was associated to younger age (COR = 1.80; 95% CI: 1.01–3.22), having other chronic illness (COR = 2.36; 95% CI = 1.20–4.63) and positive lymph node status (COR = 2.30, 95% CI: 1.28–4.13). In multivariate analysis, patients with positive lymph node status were 2.39 times greater risk to developed psychological distress (AOR = 2.39; 95% CI: 1.26–4.57), and patients with another chronic illness were 2.78 times more risk to have psychological distress (AOR = 2.78; 95% CI: 1.32–5.85) (Table 4).

## Discussion

In the Moroccan context, this study is unique for two reasons: it is the first to investigate psychological distress among BC patients with LABC, in contrast to some studies that involve all types of BC. Additionally, this study was conducted only during the diagnostic period and up to the last day before receiving neoadjuvant chemotherapy, and most studies have focused on BC r survivors or during treatment.

A diagnosis of BC is frequently followed by depression and anxiety, and finding ways to detect those who may be at risk of psychological distress is crucial [55, 56]. Therefore, the present study examines prevalence and associated variables of psychological distress among patients recently have been received diagnosis of LABC. The term psychological distress is generally defined as depression and anxiety. For this reason, one of the popular scales used widely in clinical practices of psychological distress in cancer patients was applied. Depression was measured by subscale HADS-D and cutoff greater than or equal to 8, whereas anxiety was measured by subscale HADS-A and cutoff greater than or equal to 10. In addition, we categorized participants based on the total HADS score with a cutoff of 15.

A key finding of this study was the high rate of psychological distress among participants. Quite alarmingly, more than half fell above the depression and HADS T threshold, 59.81% and 64.59% respectively, while 47.8% of our sample experienced anxiety. Our results remain higher, but near to the rates showed in some studies investigating psychological distress in patient undergoing NACT. To illustrate, a Korean prospective study [57] found that depression and anxiety was respectively 40.2% and 48% in a sample of 184 BC patients. The results of a recent prospective study conducted on 203 Koreans with BC who received NACT indicated that 35% of patients were depressed, while 34% were anxious [58]. While, a Canadian prospective study [59] of 203 BC patients undergoing NACT reported that 54.2% had high levels of anxiety at baseline. Using the Distress Thermometer, an American study [60] showed that psychological distress had reached its peak before NACT in more than half of 252 women diagnosed with non-metastatic BC and before onset NACT. With regard to other types of BC and patients outside of NACT, a meta-analysis of 39 quantitative papers, after the diagnosis of BC, the incidence of clinically significant symptoms of depression was 20%, anxiety 34%, and 39% for distress. In Morocco, one cross sectional study [61], conducted on patients with BC in different steps of cancer treatment, mentioned that 87% of them suffered from an anxiety-depressive syndrome. Interestingly, the prevalence rates found in this study are similar to those found in a study [62] of systemic lupus erythematosus patients with depression and anxiety. As with our study, this was conducted in the same hospital and shown that prevalence of depression and anxiety was respectively 57.4% (95% CI: 47.8-67%) and 55.4% (95% CI: 45.8-65%). Based on the results of various longitudinal studies [63, 64] of BC patients undergoing NACT, we anticipate that psychological distress will decrease in patients participating in our research during and at the end of NACT.

Considering the high prevalence of psychological distress revealed in the present study, it was imperative to explore the factors explaining these rates. Thus, results of this study based on multivariate analysis, revealed that younger age (less than 50) was associated with depression and anxiety. Moreover, univariate analysis confirmed that it was also related to psychological distress. The same result was found by LeVasseur et al. who indicated that patients in the high-anxiety cohort at initial oncology consultation were significantly younger compared with those in the low anxiety cohort [56]. Younger age was also the main factor associated with psychological distress in the results of a systematic review on the predictors of psychological distress in female BC survivors [65].

The second factor that showed an association with psychological distress in this study was the full health insurance coverage. In fact, the risk of de.

pression for patients having this type of insurance was greater than those with partial insurance. The explanation of this result was that the beneficiaries of full medical insurance in Morocco are generally the poor and socioeconomically vulnerable patients who were often unable to pay even for basic necessities such as food and transportation.

For clinical variables, anxiety was more prevalent in patients with BC on the right side. This can be explained by the fear of patients who had right BC before ongoing NACT to suffer mainly lymphedema in the right arm which can occur after surgery and lymph node dissection, knowing well that right-handed people are predominant with a rate of 90% among the human population. These patients can experience this fear when they meet other patients in the waiting rooms who complain of this problem or when they get information about lymphedema from other resources. This leads to them believing that they will have functional problems immediately following surgery of right BC [66].

Concerning positive lymph node status, it was statistically associated to psychological distress. In a paper exploring mental health of invasive BC patients [22], positive lymph nodes were more likely to have depression and anxiety. A similar result was shown in the study of Ilic [67], which also confirmed that patients who had BC with positive lymph nodes experienced more depression.

Chronic illness was another determinant of psychological distress in our finding. In fact, significant increases in depression and/or anxiety were associated with heart disease, arthritis, asthma, stroke, chronic neck or back pain, and hypertension [67]. Being diagnosed with BC would therefore raise the suffering of patients with these chronic diseases, especially since they will need more radiological and biological examinations, and will be subjected to more and more different medical consultations.

As far as we know, this is the first study to investigate prevalence and determinants of psychological distress, depression, and anxiety in LABC Moroccan patients. The prevalence levels of these variables remain high. Considering the finding of this study, it would be helpful to predict which patients are at risk of developing psychological diseases with a diagnosis of LABC, in order to prevent them. As we assume as well that the establishment of interventions which consider the determinants associated with the psychological variables can help in better managing the alteration of psychological features and to properly prepare Moroccan patients to start NACT. In the routine practice of health professionals, these psychological problems must be assessed by using appropriate tools, such as HADS. Numerous publications [68–75] have also highlighted the importance of nursing practices, not only in assessing psychological states but also in implementing psychological interventions.

In this sense, our cohort research project finds its relevance in observing the trajectory of psychological distress during and after NACT, after surgery, and up to 5 years after. Through such a study, it is possible to conclude the psychological state of patients and the factors influencing it, as well as the likelihood of survival. We strongly urge, however, that a research study should be conducted to design and evaluate psychological intervention programs for patients with LABC.

For proper interpretation of the results, some limitations of this study must be mentioned. The data used in our study was based on The HADS which may have led to bias, however, this scale has previously been proven reliable valid. Additionally, possibly due to our sample size, we found our results to be underpowered, while a larger sample size may have provided robust results. This finding could be replicated and validated in other Moroccan clinical settings with a variety of sample populations.

## Conclusions

It is important for people receiving neoadjuvant chemotherapy to talk to their doctor, their nurses or a mental health professional about any mental health issues they may be having. Treatment for psychological distress can include medication, psychotherapy, or both. It is important to get treatment as soon as possible to avoid further complications, especially problems of adherence to NACT or generating psychiatric diseases.

In order to identify the main changes in biopsychosocial variables over time, it is necessary to follow the patients included in our project cohort study. Following diagnosis, these patients receive a variety of treatments, including NACT sessions, target therapy, surgery, radiotherapy, and hormonal therapy. Additionally, they perform numerous routine examinations and specialized radiological and biological tests. We believe that it is crucial to develop a global model to explain their psychological adjustment. In this way, we would be able to meet patients’ needs, improve their safety and quality of care, and promote behavioral epidemiology research.

### Electronic supplementary material

Below is the link to the electronic supplementary material.

**Additional file 1: Supplementary Table S1**. Bivariate analysis of depression, anxiety and psychological distress with sociodemographic variables.

## Data Availability

The datasets used and/or analysed during the current study available from the corresponding author on reasonable request.

## Abbreviations

χ2: Chi-square
AOR: Adjusted Odds Ratio
BC: Breast cancer
CI: Confidence interval
COR: Crude Odd Ratio
ER: Estrogen receptor
HADS: Hospital Anxiety and Depression Scale
HER2: Human epidermal growth factor receptor-2
LABC: Locally advanced breast cancer
LMIC: Low and middle-income countries
NACT: Neoadjuvant chemotherapy
OR: Odds Ratio
PCR: Pathological complete response
PR: Progesterone receptor
SBR: Scarff-Bloom and Richardson
SD: Standard deviation
TNM: Tumor-Node-Metastasis
