# Supplementary material for: Evaluation of psychological distress is essential for patients with locally advanced breast cancer prior to neoadjuvant chemotherapy: baseline findings from cohort study
Source: BMC Womens Health. 2023 Aug 23;23:445. doi: 10.1186/s12905-023-02571-1 (PMC10464406; doi:10.1186/s12905-023-02571-1)
Supplement: Supplementary file 1 — Additional file 1: Supplementary Table S1. Bivariate analysis of depression, anxiety and psychological distress with sociodemographic variables. [file 12905_2023_2571_MOESM1_ESM.docx]

**Additional fil 1:**

**Supplementary Table S1.** Bivariate analysis of depression, anxiety and psychological distress with sociodemographic variables.

| **Sociodemographic variables** | **Anxiety** | | **ꭓ2^1^** | **OR^2^ (95% CI^3^)** | **Depression** | | **ꭓ2** | **OR (95% CI)** | **Psychological distress** | | **ꭓ2** | **OR (95% CI)** |
| --- | --- | --- | --- | --- | --- | --- | --- | --- | --- | --- | --- | --- |
|  | **No (%)** | **Yes (%)** |  |  | **No (%)** | **Yes (%)** |  |  | **No (%)** | **Yes (%)** |  |  |
| **Age at diagnosis** |  |  | 3.6 | 0.6  (0.3-1.0) |  |  | 2.3 | 0.6  (0.3-1.1) |  |  | 3.99* | 0.55  (0.31-0.99) |
| < 50 | 28.7 | 32.5 |  |  | 22.1 | 38.9 |  |  | 18.2 | 43.1 |  |  |
| $\geq$ 50 | 23.4 | 15.3 |  |  | 18.3 | 20.7 |  |  | 16.7 | 22.0 |  |  |
| **Marital status** |  |  | 0.94 | 0.74  (0.40-1.35) |  |  | 0.01 | 0.96  (0.5-1.8) |  |  | 0.29 | 0.84  (0.45-1.56) |
| Married | 35.4 | 16.7 |  |  | 28.4 | 42.3 |  |  | 23.9 | 46.9 |  |  |
| Single | 35.4 | 12.4 |  |  | 12.0 | 17.3 |  |  | 11.0 | 18.2 |  |  |
| **Education** |  |  | 0.11 | 1.10  (0.62-1.93) |  |  | 0.07 | 0.92  (0.52-1.64) |  |  | 0.87 | 0.75  (0.42-1.35) |
| Illiterate | 33.5 | 29.7 |  |  | 25.0 | 38.0 |  |  | 20.6 | 42.6 |  |  |
| Educated | 18.7 | 18.2 |  |  | 15.4 | 21.6 |  |  | 14.4 | 22.5 |  |  |
| **Profession** |  |  | 0.14 | 0.85  (0.37-1.92) |  |  | 0.002 | 0.98  (0.43-2.23) |  |  | 0.46 | 0.75  (0.32-1.71) |
| Unemployed | 45.0 | 42.1 |  |  | 35.1 | 51.9 |  |  | 29.7 | 57.4 |  |  |
| Employed/ | 7.2 | 5.7 |  |  | 5.3 | 7.7 |  |  | 5.3 | 7.7 |  |  |
| **Health insurance coverage** |  |  | 1.04 | 0.64  (0.27-1.49) |  |  | 2.87 | 0.48  (0.20-1.13) |  |  | 2.96 | 0.48  (0.21-1.11) |
| Total | 44.5 | 43.1 |  |  | 33.7 | 54.3 |  |  | 28.7 | 58.9 |  |  |
| Partial | 7.7 | 4.8 |  |  | 6.7 | 5.33 |  |  | 6.2 | 6.2 |  |  |
| **Residency** |  |  | 0.76 | 0.77  (0.43-1.38) |  |  | 0.08 | 1.09  (0.60-1.96) |  |  | 0.19 | 0.87  (0.47-1.61) |
| Rural | 15.3 | 16.7 |  |  | 13.5 | 18.8 |  |  | 10.5 | 21.5 |  |  |
| Urbain | 36.8 | 31.1 |  |  | 26.9 | 40.9 |  |  | 24.4 | 43.5 |  |  |
| **Ethnicity** |  |  | 0.12 | 0.89  (0.47-1.67) |  |  | 1.70 | 1.52  (0.80-2.86) |  |  | 0.07 | 1.09  (0.57-2.11) |
| Amazigh | 12.4 | 12.4 |  |  | 12.0 | 13.0 |  |  | 9.1 | 15.8 |  |  |
| Arabic | 39.7 | 35.4 |  |  | 28.4 | 46.6 |  |  | 25.8 | 49.3 |  |  |
| **Patient's monthly income** |  |  | 0.00 | 0.99  (0.42-2.37) |  |  | 0.10 | 0.86  (0.36-2.08) |  |  | 0.20 | 0.81  (0.33-1.98) |
| No income | 46.4 | 42.6 |  |  | 35.6 | 53.4 |  |  | 30.6 | 58.4 |  |  |
| 250 $ | 5.7 | 5.3 |  |  | 4.8 | 6.3 |  |  | 4.3 | 6.7 |  |  |
| **Monthly family income** |  |  | 7.87** |  |  |  | 5.61 |  |  |  | 3.03 |  |
| No income | 4.9 | 10.7 |  |  | 3.4 | 12.3 |  |  | 3.4 | 12.2 |  |  |
| < 250 $ | 41.5 | 30.2 |  |  | 31.9 | 39.7 |  |  | 27.3 | 44.4 |  |  |
| ≥ 250 $ | 5.9 | 6.8 |  |  | 5.4 | 7.4 |  |  | 4.4 | 8.3 |  |  |
| **Nomber of childern** |  |  | 0.41 | 0.82  (0.45-1.49) |  |  | 2.00 | 0.64  (0.34-1.18) |  |  | 1.43 | 0.67  (0.36-1.28) |
| <3 | 21.7 | 22.9 |  |  | 14.9 | 29.7 |  |  | 13.1 | 31.4 |  |  |
| ≥ 3 | 29.7 | 25.7 |  |  | 24.7 | 31.0 |  |  | 21.1 | 34.3 |  |  |
| **Having children** |  |  | 1.26 | 0.56  (0.21-1.54) |  |  | 2.55 | 0.40  (0.12-1.27) |  |  | 0.008 | 0.95  (0.34-2.68) |
| No | 4.0 | 6.3 |  |  | 2.3 | 8.0 |  |  | 3.4 | 6.9 |  |  |
| Yes | 47.4 | 42.3 |  |  | 37.4 | 52.3 |  |  | 30.9 | 58.9 |  |  |

* *p* ≤ 0.05 ; ** *p* ≤ 0.01 ; *** *p* ≤ 0.001, ^1^ Chi-square, **^2^** Odds Ratio, **^3^** Confidence interval

**Supplementary Table S2** Bivariate analysis of depression, anxiety and psychological distress with clinical variables

| C**linical variables** | **Anxiety** | | **ꭓ2** | | **OR**  **(95% CI)** | | **Depression** | | | | **ꭓ2** | | **OR**  **(95% CI)** | | **Psychological distress** | | | | | **ꭓ2** | | | **OR**  **(95% CI)** | | |  |
| --- | --- | --- | --- | --- | --- | --- | --- | --- | --- | --- | --- | --- | --- | --- | --- | --- | --- | --- | --- | --- | --- | --- | --- | --- | --- | --- |
|  | **No (%)** | **Yes (%)** | |  | |  | | **No**  **(%)** | **Yes (%)** | | |  | |  | | **No**  **(%)** | | **Yes (%)** | | |  | | |  | |  |
| **Breast Tumor Laterality** |  |  | | 6.67** | | 0.48  (0.27-0.84) | |  |  | | | 0.25 | | 1.15  (0.66-2.00) | | |  | |  | | | 2.76 | | | 0.61  (0.34-1.09) | |
| Right | 22.5 | 29.2 | |  | |  | | 21.6 | 29.8 | | |  | |  | | | 15.3 | | 36.4 | | |  | | |  | |
| Left | 29.7 | 18.7 | |  | |  | | 18.8 | 29.8 | | |  | |  | | | 19.6 | | 28.7 | | |  | | |  | |
| **Menopause status** |  |  | | 0.58 | | 0.80  (0.47-1.39) | |  |  | | | 0.71 | | 0.78  (0.45-1.37) | | |  | |  | | | 0.60 | | | 0.79  (0.45-1.41) | |
| Premenopausal | 24.9 | 25.4 | |  | |  | | 18.8 | 31.3 | | |  | |  | | | 16.3 | | 34.0 | | |  | | |  | |
| Postmenopausal | 27.3 | 22.5 | |  | |  | | 21.6 | 28.4 | | |  | |  | | | 18.7 | | 31.1 | | |  | | |  | |
| **Time from finding out the symptoms** |  |  | | 0.26 | |  | |  |  | | | 0.38 | |  | | |  | |  | | | 0.54 | | |  | |
| < 6 months | 22.7 | 21.7 | |  | |  | | 17.5 | 27.2 | | |  | |  | | | 14.5 | | 30.0 | | |  | | |  | |
| Between 6 and 12 months | 14.5 | 12.1 | |  | |  | | 10.7 | 16.0 | | |  | |  | | | 10.1 | | 16.4 | | |  | | |  | |
| ≥ 12 months | 14.5 | 14.5 | |  | |  | | 12.6 | 16.0 | | |  | |  | | | 10.6 | | 18.4 | | |  | | |  | |
| **Family cancer history** |  |  | | 0.10 | | 0.88  (0.41-1.89) | |  |  | | | 0.03 | | 0.92  (0.42-2.01) | | |  | |  | | | 0.005 | | | 0.97  (0.43-2.15) | |
| No | 44.0 | 41.1 | |  | |  | | 34.1 | | 51.0 | |  | |  | | | 29.7 | | 55.5 | | |  | | |  | |
| Yes | 8.1 | 6.7 | |  | |  | | 6.3 | | 8.7 | |  | |  | | | 5.3 | | 9.6 | | |  | | |  | |
| **Chronic illness** |  |  | | 2.21 | | 0.60  (0.30-1.17) | |  | |  | | 5.34* | | 0.45  (0.23-0.89) | | |  | |  | | | 6.46** | | | 0.42  (0.21-0.82) | |
| No | 38.9 | 39.4 | |  | |  | | 28.5 | | 49.8 | |  | |  | | | 24.0 | | 54.3 | | |  | | |  | |
| Yes | 13.5 | 8.2 | |  | |  | | 12.1 | | 9.7 | |  | |  | | | 11.1 | | 10.6 | | |  | | |  | |
| **Histological type** |  |  | | 0.91 | | 0.57  (0.18-1.79) | |  | |  | | 0.04 | | 0.88  (0.29-2.65) | | |  | |  | | | 0.45 | | | 0.68  (0.22-2.06) | |
| Ductal | 47.6 | 45.7 | |  | |  | | 37.2 | | 56.0 | |  | |  | | | 31.7 | | 61.5 | | |  | | |  | |
| Lobular and others | 4.3 | 2.4 | |  | |  | | 2.9 | | 3.9 | |  | |  | | | 2.9 | | 3.8 | | |  | | |  | |
| **Tumour size** |  |  | | 0.006 | | 1.02  (0.58-1.78) | |  | |  | | 1.85 | | 1.48  (0.84-2.61) | | |  | |  | | | 0.00 | | | 1.00  (0.56-1.80) | |
| T2/T3 | 20.1 | 18.2 | |  | |  | | 17.8 | | 20.7 | |  | |  | | | 13.4 | | 24.9 | | |  | | |  | |
| T4 | 32.1 | 29.7 | |  | |  | | 22.6 | | 38.9 | |  | |  | | | 21.5 | | 40.2 | | |  | | |  | |
| **Lymph node status** |  |  | | 3.77 | | 1.75  (0.99-3.09) | |  | |  | | 4.26* | | 1.82  (1.02-3.22) | | |  | |  | | | 7.92** | | | 2.30  (1.28-4.13) | |
| Negative | 23.0 | 14.8 | |  | |  | | 18.8 | | 19.2 | |  | |  | | | 17.7 | | 20.1 | | |  | | |  | |
| Positive | 29.2 | 33.0 | |  | |  | | 21.6 | | 40.4 | |  | |  | | | 17.2 | | 45.0 | | |  | | |  | |
| **ER**^2^ |  |  | | 1.42 | | 0.71  (0.40-1.24) | |  | |  | | 2.48 | | 0.62  (0.35-1.12) | | |  | |  | | | 3.01 | | | 0.58  (0.31-1.06) | |
| Negative | 18.4 | 21.4 | |  | |  | | 13.2 | | 26.8 | |  | |  | | | 10.7 | | 29.1 | | |  | | |  | |
| Positive | 33.0 | 27.2 | |  | |  | | 26.3% | | 33.7 | |  | |  | | | 23.3 | | 36.9 | | |  | | |  | |
| **PR**^3^ |  |  | | 1.75 | | 0.69  (0.40-1.19) | |  | |  | | 1.56 | | 0.70  (0.39-1.22) | | |  | |  | | | 3.81 | | | 0.56  (0.31-1.01) | |
| Negative | 23.7 | 26.6 | |  | |  | | 18.0 | | 32.5 | |  | |  | | | 14.0 | | 36.2 | | |  | | |  | |
| Positive | 28.0 | 21.7 | |  | |  | | 21.8 | | 27.7 | |  | |  | | | 20.3 | | 29.5 | | |  | | |  | |
| **HER2**^4^ |  |  | | 2.96 | | 0.59  (0.33-1.07) | |  | |  | | 0.38 | | 0.83  (0.46-1.49) | | |  | |  | | | 0.79 | | | 0.76  (0.41-1.38) | |
| Negative | 32.0 | 34.5 | |  | |  | | 25.9 | | 40.5 | |  | |  | | | 21.8 | | 44.7 | | |  | | |  | |
| Positive | 20.4 | 13.1 | |  | |  | | 14.6 | | 19.0 | |  | |  | | | 13.1 | | 20.4 | | |  | | |  | |
| **KI 67** |  |  | | 0.002 | | 0.98  (0.42-2.28) | |  | |  | | 0.07 | | 0.88  (0.37-2.08) | | |  | |  | | | 0.00 | | | 1.00  (0.41-2.40) | |
| < 20 | 6.9 | 6.3 | |  | |  | | 5.3 | | 8.0 | |  | |  | | | 4.8 | | 8.5 | | |  | | |  | |
| ≥ 20 | 45.5 | 41.3 | |  | |  | | 37.2 | | 49.5 | |  | |  | | | 31.2 | | 55.6 | | |  | | |  | |
| **Molecular subtype** |  |  | | 7.06 | |  | |  | |  | | 6.47 ^1^ | |  | | |  | |  | | | 12.14* | | |  | |
| Luminal A | 2.5 | 3.5 | |  | |  | | 1.5 | | 4.6 | |  | |  | | | 1.0 | | 5.1 | | |  | | |  | |
| Luminal B | 19.7 | 16.2 | |  | |  | | 17.3 | | 18.3 | |  | |  | | | 16.2 | | 19.7 | | |  | | |  | |
| Luminal B HER2 | 12.6 | 8.1 | |  | |  | | 9.6 | | 11.2 | |  | |  | | | 8.6 | | 12.1 | | |  | | |  | |
| Her2 Enriched | 7.6 | 4.5 | |  | |  | | 4.1 | | 8.1 | |  | |  | | | 4.0% | | 8.1 | | |  | | |  | |
| TNBC^6^ | 9.6 | 15.7 | |  | |  | | 7.6 | | 17.8 | |  | |  | | 4.5% | | 20.7 | | |  | | |  | |  |

* *p* ≤ 0.05 ; ** *p* ≤ 0.01 , ^1^ Fisher’s exact test, ^2^ Estrogen receptor; ^3^ Progesterone receptor, ^4^ HER2 = human epidermal growth factor receptor 2, ^5^TNBC = Triple-negative Breast Cancer
